# *Salmonella* effector SpvB aggravates dysregulation of systemic iron metabolism via modulating the hepcidin−ferroportin axis

**DOI:** 10.1080/19490976.2020.1849996

**Published:** 2021-01-21

**Authors:** Qifeng Deng, Sidi Yang, Lanqing Sun, Kedi Dong, Yuanyuan Li, Shuyan Wu, Rui Huang

**Affiliations:** Department of Medical Microbiology, School of Biology & Basic Medical Sciences, Medical College of Soochow University, Suzhou, Jiangsu, PR China

**Keywords:** *Salmonella*, SpvB; systemic iron metabolism, hepcidin–ferroportin axis, TREM-1

## Abstract

Iron withholding, an essential component of nutritional immunity, plays a fundamental role in host resistance to *Salmonella* infection. Our previous study showed that SpvB, an important pSLT-encoded cytotoxic effector, facilitated *Salmonella* pathogenesis within macrophages via perturbing cellular iron metabolism. However, the underlying mechanisms of SpvB in *Salmonella*-relevant disorders of systemic iron metabolism have not yet been identified. Here, we demonstrated that SpvB facilitated *Salmonella* to scavenge iron from the host by modulating the hepcidin–ferroportin axis, a key regulator of systemic iron metabolism. We observed that SpvB enhanced hepatic hepcidin synthesis in a STAT3-dependent manner, but not the BMP/SMAD pathway. This subsequently resulted in a reduction of the unique cellular iron exporter ferroportin, which facilitated hypoferremia and hepatic iron accumulation and ultimately countered the limitation of iron availability, thereby improving the chances of *Salmonella* survival and replication. Moreover, SpvB promoted the production of proinflammatory molecules associated with the infiltration of inflammatory cells via highly upregulating TREM-1 signaling. Our data supported a role of TREM-1 in SpvB-related dysregulation of host iron metabolism and suggested that targeting TREM-1 might provide a potential therapeutic strategy to prevent or alleviate *Salmonella* pathogenesis.

## Introduction

*Salmonella enterica* serovar Typhimurium (*S. typhimurium*) is a facultative intracellular gram-negative bacterial pathogen that causes severe gastroenteritis and systemic inflammation in both humans and animals. Approximately 180 million cases, or 9% of the diarrheal cases involving this bacterium, are reported annually around the world.^1^ As one of most prevalent food- and water-borne pathogens, *S. typhimurium* gains access to the gastrointestinal barrier, which is associated with diarrhea and acute inflammation, then disseminates to the mesenteric lymph nodes, and eventually spreads to and colonizes other extra-intestinal tissues, such as the liver, during the late stage of infection.^2^

*S. typhimurium* has evolved several mechanisms to counteract the host antimicrobial defense. The *Salmonella* plasmid virulence (*spv*) locus is a highly conserved region on the pSLT virulence plasmid from *Salmonella* strains of clinically significant serovars, e.g., *S. typhimurium, S. enteritidis, S. choleraesuis, S. dublin*, and *S. arizona, et al*.^3^ The SpvB effector, which is one of the *spv* locus-encoded cytotoxic proteins, has been reported to participate in several facets of *Salmonella* pathogenesis. For instance, after translocating from the *Salmonella*-containing vacuole (SCV) into the cytoplasm of the host cell via *Salmonella* pathogenicity island-2 (SPI-2) type III secretion system (T3SS), SpvB induces apoptotic cell death in eukaryotic cells and contributes to severe inflammatory injury in mice^4,5^ In addition to its role in promoting *Salmonella* virulence, SpvB downregulates autophagic flux via the cytotoxic effects of actin depolymerization and contributes to the intracellular replication and survival of *Salmonella*.^6^

Iron is essential for humans, as it functions as a redox catalyst for cellular enzymatic processes, and disorders of host iron homeostasis strongly influence the course and outcome of *Salmonella* infection. Iron is a necessary nutrient for nearly all bacteria, including *Salmonella* in their intracellular proliferation and survival^7,8^ In the competition for host iron, *Salmonella* obtains iron resources to meet their own nutrient requirements, which results in hypoferremia and iron accumulation in the liver and spleen.^9^ Therefore, it is significant to maintain physiological iron homeostasis during *Salmonella* infection.

There are two regulatory systems involved in iron metabolism: one that modulates cellular iron metabolism via iron-regulatory proteins and another that regulates systemic iron metabolism predominantly via the hepcidin–ferroportin (FPN) axis.^10^ Hepcidin is a defensin-like, cysteine-rich antimicrobial peptide that prevents iron release from intracellular sources by directly binding to the sole mammalian iron exporter, FPN, thereby facilitating the internalization and lysosomal degradation of FPN and ultimately sequestering the iron in cells^11,12^ Hepcidin is transcriptionally regulated by iron and inflammatory cytokines through the BMP/SMAD and JAK/STAT3 signaling pathways respectively^13,14^ In recent years, a growing number of experiments have indicated that enhanced hepcidin production during *Salmonella* infection contributes to bacterial growth and virulence. Despite this, however, the potential mechanism of how *Salmonella* modulates the hepcidin–FPN axis and subsequently responds to host iron withdrawal remains poorly understood. Previous work from our laboratory reported a novel contribution of SpvB to *Salmonella* pathogenesis by disturbing cellular iron metabolism. SpvB has been shown to inhibit FPN expression at the transcriptional level via a proteasome pathway dependent upon NRF2 reduction, thereby decreasing iron efflux and increasing intracellular iron concentration.^15^ In this work, we aim to expand our understanding on the effect of SpvB *in vivo* by investigating the hypothesis that SpvB influences systemic iron metabolism in a hepcidin-dependent pathway.

## Material and methods

### Animal and ethics statement

C57BL/6 mice were obtained from the experimental animal center of Soochow University. Hepcidin (*Hamp*) heterozygous mice (on a C57BL/6 J genetic background) were a gift from Professor Youjia Xu (The Second Affiliated Hospital of Soochow University, China).^16^ The generation and genotype identification of mice deficient in *Hamp* has been described previously. Mice were bred locally under specific-pathogen-free conditions in individually ventilated cages and received sterile water and food *ad libitum*. Eight- to ten- weeks old mice were used in this study. All animal experiments were approved by the Animal Experimental Committee of Soochow University (Grant 2111270) and complied with the National Institutes of Health Guidelines for the Care and Use of Laboratory Animals (NIH Guidelines).

### Bacterial strains and growth condition

Wild-type (WT) *S. typhimurium* strain SL1344 was a gift from Professor Qian Yang (Nanjing Agricultural University, Nanjing, China). The *spvB* deletion mutant strain SL1344-*ΔspvB* and the *spvB* complemented strain SL1344-c-*spvB* were constructed in our previous work.^15^ WT and *ΔspvB* strains were grown on Luria-Bertani (LB) agar (Hangwei, Hangzhou, China) plates or in LB medium (Hangwei), and c-*spvB* strain was grown in LB broth containing 100 μg/ml ampicillin (Sangon Biotech, Shanghai, China).

### S. typhimurium infection in vivo

*Salmonella* strains were inoculated in LB medium overnight. On the day of experiments, bacterial cultures were diluted in a ratio of 1:100 and then agitated for a further 3 h. Upon washing 3 times with PBS, fresh bacterial suspension was ready for the infection experiment. Following orally administrated with 20 mg/mouse streptomycin (Sangon Biotech) for 24 h, mice were orally infected with 1 × 10^7^ colony-forming units (CFUs) of SL1344 or *ΔspvB* strain. Samples were collected at indicated time points. Hepatic bacterial burden was quantified by plating homogenized hepatic tissues in appropriate dilutions on *Salmonella-Shigella* agar plates (Hangwei). The plates were incubated overnight at 37°C and colonies were counted the next day. To investigate the role of SpvB in *Salmonella*-associated dysregulation of systemic iron metabolism, *S. typhimurium* infected mice were administered the iron chelator deferasirox (DFX) *ad libitum* orally with a solution of 500 mg DFX (Meilunbio, Beijing, China) dissolved in 1000 ml drinking water.^17^ The number of *S. typhimurium* in the liver was determined as described above. To analyze the relationship between SpvB and STAT3 pathway, mice were intraperitoneally treated with 20 mg/kg the STAT3 inhibitor Stattic as previously described,^18^ and then infected with different *S. typhimurium* strains as mentioned above. To assess the role of TREM-1 in SpvB-mediated pathogenesis, mice were intraperitoneally injected with 100 μg/mouse LP17 peptide (LQVTDSGLYRCVIYHPP), a synthetic polypeptide inhibitor of TREM-1 dimerization and activation, at 1 day post-infection.^19^

### Quantitative PCR

GGTotal RNA was isolated from mouse liver using TRIzol reagent (Beyotime Biotechnology, Shanghai, China) and reverse-transcribed using the All-in-one RT MasterMix kit (Applied Biological Materials, Richmond, BC, Canada) according to the manufacturer’s instructions. cDNAs were analyzed by the ViiA7 real-time PCR instrument (Applied Biosystems, Carlsbad, CA, USA) using EvaGreen MasterMix-Low ROX (Applied Biological Materials). Specific primer sequences were listed in Table 1. All expression levels were normalized to *Gapdh* or *β-ACTIN* expression. Values were expressed as fold induction in comparison to untreated control mice.

**Table 1.** Primer sequences for quantitative PCR

Primers		Sequences
***mHamp***	**sense**	5’-TTGCGATACCAATGCAGAAGA-3’
	**antisense**	5’-GATGTGGCTCTAGGCTATGTT-3’
***mCcl2***	**sense**	5’-GTTGGCTCAGCCAGATGCA-3’
	**antisense**	5’-AGCCTACTCATTGGGATCATCTTG-3’
***mCcl3***	**sense**	5’-ACCATGACACTCTGCAACCA-3’
	**antisense**	5’-GATGAATTGGCGTGGAATCT-3’
***mCxcl10***	**sense**	5’-CGATGGATGGACAGCAGAGAGCC-3’
	**antisense**	5’-CCATGGCTTGACCATCATCCTGCAG-3’
***mIl1β***	**sense**	5’-CTTCAGGCAGGCAGTATC-3’
	**antisense**	5’-AGCAGGTTATCATCATCATC-3’
***mIl6***	**sense**	5’-CCAGAAACCGCTATGAAGTTCCT-3’
	**antisense**	5’-CACCAGCATCAGTCCCAAGA-3’
***mTnfα***	**sense**	5’-GCCTCTTCTCATTCCTGCTTG-3’
	**antisense**	5-CTGATGAGAGGGAGGCCATT-3’
***mTrem-1***	**sense**	5’-CGCCTGGTGGTGACCAAGGG-3’
	**antisense**	5’-ACAACCGCAGTGGGCTTGGG-3’
***mGapdh***	**sense**	5’-AGGTCGGTGTGAACGGATTTG-3’
	**antisense**	5’-TGTAGACCATGTAGTTGAGGTCA-3’
***hHAMP***	**sense**	5’-CACAACAGACGGGACAAC-3’
	**antisense**	5’-CGCAGCAGAAAATGCAGA-3’
***hβ-ACTIN***	**sense**	5’-ATTGCCGACAGGATGCAGAA-3’
	**antisense**	5’-GCTGATCCACATCTGCTGGAA-3’

m, mouse; h, human

### Western blot analysis

Liver tissues were lysed in RIPA Lysis Buffer (Beyotime Biotechnology) containing a protease and phosphatase inhibitor, and protein concentrations were measured using the BCA Protein Assay (Beyotime Biotechnology). Protein extracts were separated on 12% gels and then electroblotted onto PVDF membranes (MilliporeSigma, Burlington, MA, USA). After blocking nonspecific binding with 5% nonfat dry milk powder in TBST, membranes were probed with primary antibodies overnight at 4°C and incubated with the appropriate HRP-labeled secondary antibodies at room temperature for 1 h. Membranes were then visualized with an enhanced chemiluminescence luminescence reagent (Meilunbio). The intensities of the bands were calculated by ImageJ Launcher broken symmetry software program (NIH, Bethesda, MD, USA). Antibodies to the following proteins were used: FPN (sc-49668; Santa Cruz Biotechnology, Dallas, TX, USA) for *in vivo* experiments; FPN (NBP1-21502; Novus Biologicals, Littleton, CO, USA) for *in vitro* experiments; Phospho-STAT3 (Tyr705) (YP0251; ImmunoWay, Plano, TX, USA); Phospho-Smad1 (Ser463/465)/Smad5 (Ser463/465)/Smad9 (Ser465/467) (D5B10) (13820; Cell Signaling Technology, Danvers, MA, USA); TREM-1 (MAB1187; R&D Systems, Minneapolis, MN, USA); IL6 (PB0060; Boster Biological Technology, Pleasanton, CA, USA); α-Tubulin (AF0001; Beyotime Biotechnology); β-Actin (bs-0061 R; Bioss, Woburn, MA, USA); and GAPDH (bs-10900 R; Bioss).

### Isolation of liver non-parenchymal cells and flow cytometry analysis

Liver non-parenchymal cells were isolated as previously described.^20^ In brief, livers were harvested, perfused with calcium and magnesium-free Hank’s Balanced Salt Solution (HBSS; Beyotime Biotechnology), and digested with collagenase type Ι (Worthington, Lakewood, NJ, USA). The cell suspension was filtered through a 70 μm cell strainer (Sorfa, Zhejiang, China), centrifuged to eliminate hepatocytes, and then eliminated erythrocyte the Red Blood Cell Lysis Buffer (Beyotime Biotechnology) according to the manufacturer’s instructions. The non-parenchymal cells were incubated with different antibodies at 4°C for 30 min. Antibodies were used as follows: F4/80 (123108; BioLegend, San Diego, CA, USA); CD11b (101212; BioLegend); FITC Rat IgG2a κ (400505; BioLegend) and APC Rat IgG2b κ (400611; BioLegend).

### Iron measurement in the serum and liver

Blood samples collected from anesthetized mice through intracardiac puncture. Livers were harvested, weighed, mechanically homogenized in RIPA Lysis Buffer and mixed with HCl (0.01 M final concentration). Iron content was detected using an iron assay kit (BioAssay Systems, Hayward, CA, USA) according to the manufacturer’s instructions.

### Histopathologic analysis and Perls’ Prussian Blue staining

Liver samples were fixed in 4% paraformaldehyde, embedded in paraffin and cut into 5-μm thick sections. To show inflammatory lesions, sections were subjected to hematoxylin-eosin (H&E) staining (Servicebio, Wuhan, China). To assess tissue iron concentration, sections were incubated in the Perls’ Prussian Blue working solution (Servicebio) at 60°C for 30 min and subsequently stained with nuclear fast red at room temperature for 5 min. All photomicrographs were taken with a Nikon Eclipse Ni-U fluorescence microscope (Nikon Corporation, Tokyo, Japan) with NIS-Elements F (Nikon Corporation).

### Cell culture

HepG2 cells (human hepatoma cell line) were kindly provided by Professor Chengliang Gong (Soochow University, Suzhou, China). These cells were maintained in DMEM (HyClone Laboratories, Logan, UT, USA) containing 10% (v/v) heat-inactivated fetal bovine serum (FBS; Biological Industries, Kibbutz Beit-Haemek, Israel). THP-1 cells (human monocytic cell line), a gift from Professor Yanyun Zhang (Shanghai Institute of Nutrition and Health, Shanghai, China), were grown in RPMI 1640 (HyClone Laboratories) supplemented with 10% (v/v) heat-inactivated FBS.

### Salmonella infection in vitro

The *Salmonella* suspension was prepared as described above. Following 1 h of incubation with bacteria, cells were washed 3 times with PBS and then placed in fresh medium containing 10% (v/v) heat-inactivated FBS and 100 μg/ml amikacin (MilliporeSigma). After 2 h, the cell culture medium was replaced with fresh medium containing 10% (v/v) heat-inactivated FBS and 10 μg/ml amikacin (MilliporeSigma).

### Co-culture and treatment

THP-1 cells were differentiated into adherent macrophage-like cells by 100 nM phorbol 12-myristate 13-acetate (PMA; MilliporeSigma) for 48 h. A non-contact culture system was set up following a published protocol^21,22^ In brief, THP-1 macrophages were seeded at 5 × 10^5^ cells per well on the top surface of the Transwell inserts (Corning-Costar, Corning, NY, USA). HepG2 cells were grown in 6-well plates. On the day of infection experiments, HepG2 cells were overlaid with Transwell inserts containing THP-1 macrophages, and the bacterial suspension was added to the apical side of the Transwell system. To assess iron content of macrophages in this co-cultured system, THP-1 macrophages were incubated with 100 μM ferrous chloride (Adamas, Shanghai, China) for 16 h prior to be placed above HepG2 cells.^22^ The relative iron concentration in co-cultured macrophages lysates was determined using an iron assay kit (BioAssay Systems).

### Transfection experiment

The HAMP small interfering RNA (siRNA), TREM-1 siRNA and negative control siRNA (NC siRNA) were purchased from GenePharma (Shanghai, China). Cells were transfected with siRNA using Lipofectamine RNAiMAX reagent (Thermo Fisher Scientific, Waltham, MA, USA) for 24 h according to the manufacturer’s instructions. Knockdown efficiency was assessed by quantitative PCR or Western blot with TREM-1 antibody (YT5133; ImmunoWay). The *spvB* overexpression plasmid pEGFP-*spvB* was constructed in our previous work.^6^ THP-1 macrophages were transiently transfected with pEGFP-*spvB* or the control vector pEGFP-N1 by Lipofectamine 3000 (Thermo Fisher Scientific) according to the manufacturer’s instructions.

### Statistics

Data are presented as mean ± SEM. Statistical analysis was performed using IBM SPSS statistics 22 (Chicago, IL, USA). Comparisons of 2 groups were analyzed using an independent Student’s *t-*test. Comparisons among multiple groups were performed with one-way ANOVA with Student-Newman-Keuls (S-N-K) correction. Values of *P* < .05 were considered statistically significant.

## Results

### *SpvB effector protein is significant to* S. typhimurium *dysregulation of host iron metabolism*

**Figure 1.**
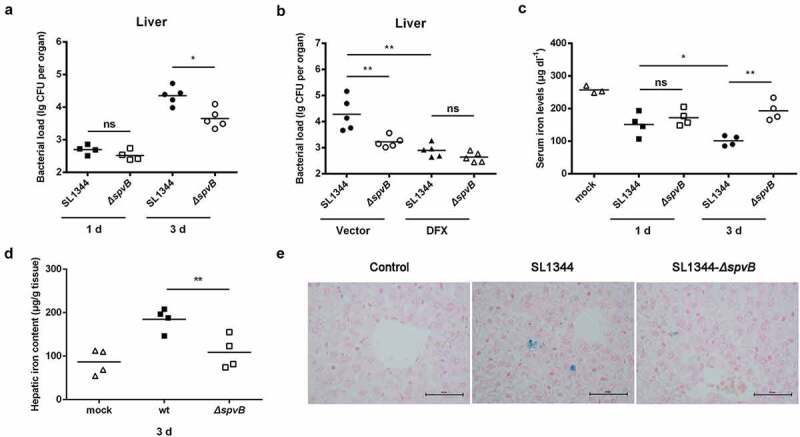
**SpvB effector protein is significant to *S. typhimurium* dysregulation of host iron metabolism**. Streptomycin-pretreated mice were orally infected with 1 × 10^7^ CFUs of either the WT or the *ΔspvB* mutant *S. typhimurium* strain. *a*) Hepatic bacterial load at 1 and 3 days post-infection was determined by plating. *b*) *S. typhimurium*-infected mice were administered either DFX dissolved in drinking water or the same volume of drinking water. Hepatic bacterial load at 3 days post-infection was determined by plating. *c*) Serum iron levels at 1 and 3 days post-infection were measured with a colorimetric assay. *d*) Hepatic iron content at 3 days post-infection was determined on the basis of a multiscan spectrum. *e*) Perls’ Prussian Blue staining of the liver at 3 days post-infection. Original magnification, ×40; scale bars: 50 μm. One of 3 representative histology experiments is shown. Statistical analysis was performed with IBM SPSS statistics 22. Data were compared with independent Student’s *t-*test. Values are expressed as the mean ± SEM, and statistically significant differences are indicated. *P< .05; **P< .01; ns, not significant

First, we assessed whether the *Salmonella* effector SpvB contributes to the hepatic *Salmonella* pathogenesis via weakening the host iron-withholding defenses. Streptomycin-pretreated mice were orally infected with either the WT *S. typhimurium* or the *ΔspvB* mutant *S. typhimurium* strain to study host-pathogen interactions. We counted the number of *Salmonella* in the liver at 1 and 3 days post-infection and found that even though there were no apparent differences between these *S. typhimurium*-infected mice at 1 day post-infection, mice infected with the WT strain contained a significantly higher hepatic bacterial burden than mice infected with the *ΔspvB* strain at 3 days post-infection (Figure 1a). We subsequently investigated whether limitation of iron availability for *S. typhimurium* might reverse this difference *in vivo. S. typhimurium*-infected mice were further orally treated with the iron chelator DFX. Our data showed that DFX not only decreased the hepatic bacterial load, but also eliminated the significant difference in *S. typhimurium* survival between mice infected with the WT and *ΔspvB* strains (Figure 1b). We further examined the changes in the host iron homeostasis in response to *S. typhimurium* infection. In agreement with our previous study, there was a significant decrease in serum iron levels in mice infected with the WT strain when compared to mice infected with the *ΔspvB* strain at 3 days post-infection (Figure 1c). Interestingly, the hepatic iron concentration in mice infected with the WT strain was significantly higher than that detected in mice infected with the *ΔspvB* strain (Figure 1d, e). These data demonstrated that SpvB is associated with *S. typhimurium* pathogenesis, at least partly through perturbing host iron metabolism.

### SpvB mediates alternation of serum and hepatic iron levels through activation of hepcidin expression

**Figure 2.**
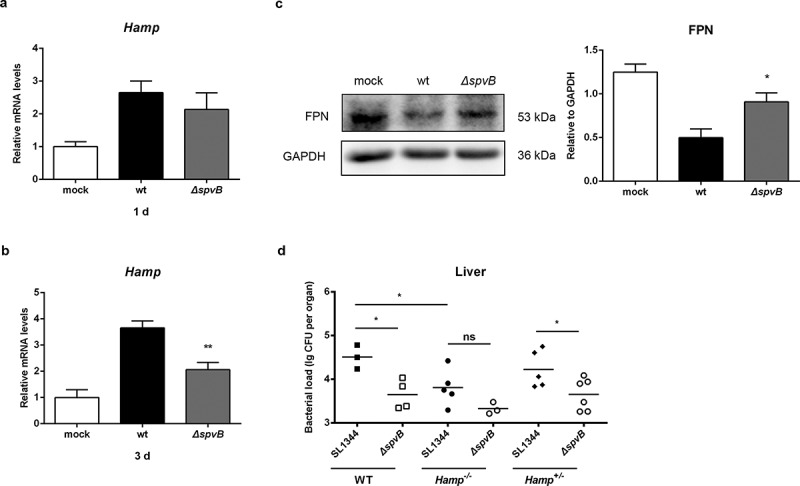
**SpvB contributes to *Salmonella*-induced disorders of systemic iron metabolism by controlling the hepcidin-FPN axis**. *a-c*) Streptomycin-pretreated mice were orally infected with 1 × 10^7^ CFUs of either the WT or the *ΔspvB* mutant *S. typhimurium* strain. Hepatic *Hamp* levels at 1 day (*a*) or 3 days (*b*) post-infection were determined by quantitative PCR (n = 3–4 mice, respectively). *c*) Western blot analysis of whole liver lysates at 3 days post-infection with specific antibodies to FPN and the control GAPDH (n = 4 mice, respectively). Densitometric analysis of FPN relative to GAPDH protein and one of 4 representative western blot experiments are shown. *d*) Streptomycin-pretreated WT, *Hamp^+/−^* and *Hamp^−/−^* mice were orally infected with 1 × 10^7^ CFUs of either the WT or the *ΔspvB* mutant *S. typhimurium* strain. Hepatic bacterial load at 3 days post-infection was determined by plating. Statistical analysis was performed with IBM SPSS statistics 22. Data were compared with independent Student’s *t-*test. Values are expressed as the mean ± SEM, and statistically significant differences are indicated. *P< .05; **P< .01; ns, not significant

The hepcidin–FPN axis is considered to be a crucial regulator of host iron homeostasis. Therefore, we hypothesized that SpvB leads to changes in hepatic hepcidin expression and eventually contributes to *Salmonella* pathogenesis. At 1 day post-infection, the mRNA transcript level of hepatic hepcidin in WT-infected mice was similar to that in *ΔspvB*-infected mice (Figure 2a). However, at 3 days post-infection, mice infected with the WT strain showed significantly higher hepcidin expression than mice infected with the *ΔspvB* strain (Figure 2b). In line with this observation, mice infected with the WT strain displayed significantly lower FPN protein levels in the liver as compared with mice infected with the *ΔspvB* strain (Figure 2c). To better understand the role of hepcidin in SpvB-mediated *Salmonella* pathogenesis and iron metabolic disorders, WT (C57BL/6 J), *Hamp* gene knockout (*Hamp^−/−^*) and heterozygous (*Hamp^+/−^*) mice were infected orally with either the WT or *ΔspvB S. typhimurium* strain. Our data showed that the liver of *Hamp^−/−^* mice contained less bacteria than those of WT and *Hamp^+/−^* mice at 3 days post-infection, suggesting that hepcidin played a role in host defense against *Salmonella* infection. Importantly, there was no apparent difference in the number of *Salmonella* in the liver of *Hamp^−/−^* mice infected with the WT strain as compared to the *ΔspvB* strain (Figure 2d). These data suggested that SpvB contributes to dysregulation of host iron metabolism via acting on the hepcidin–FPN axis.

### SpvB induces hepatic hepcidin expression in a STAT3-dependent manner

**Figure 3.**
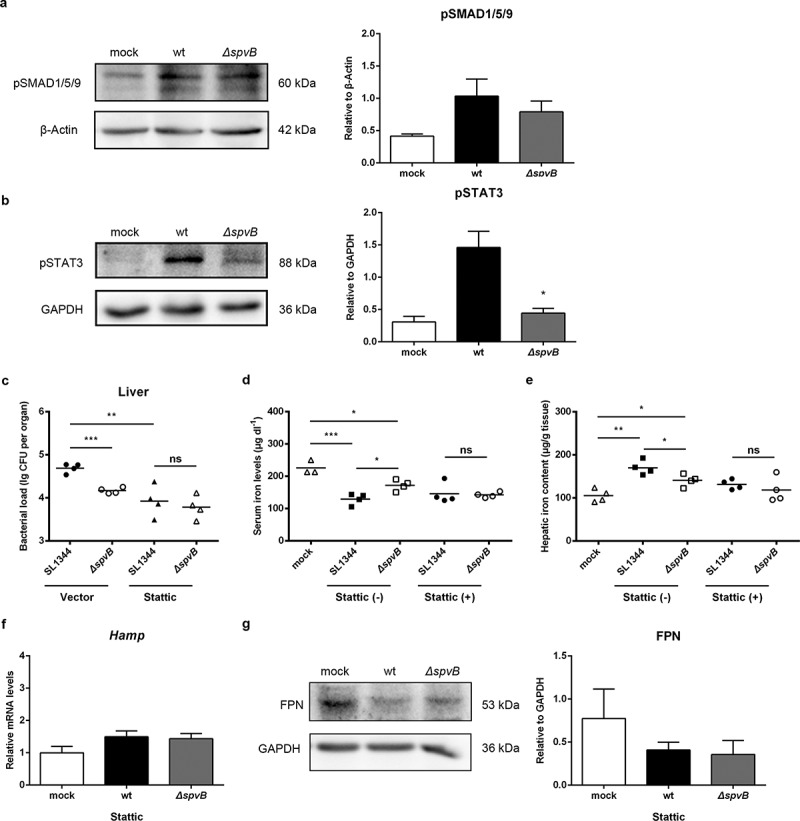
**SpvB increases hepatic hepcidin expression through the STAT3-dependent pathway**. *a, b*) Streptomycin-pretreated mice were orally infected with 1 × 10^7^ CFUs of either the WT or the *ΔspvB* mutant *S. typhimurium* strain and analyzed at 3 days post-infection. *a*) Western blot analysis of whole liver lysates with specific antibodies to pSMAD1/5/9 and the control β-Actin (n = 3 mice, respectively). Densitometric analysis of pSMAD1/5/9 relative to β-Actin protein and one of 3 representative western blot experiments are shown. *b*) Western blot analysis of whole liver lysates with specific antibodies to pSTAT3 and the control GAPDH (n = 3 mice, respectively). Densitometric analysis of pSTAT3 relative to GAPDH protein and one of 3 representative western blot experiments are shown. *c-g*) *S. typhimurium*-infected mice were administered i.p with either Stattic or the same volume of vector and analyzed at 3 days post-infection. *c*) Hepatic bacterial load was determined by plating. *d*) Serum iron levels were measured with a colorimetric assay. *e*) Hepatic iron content was determined on the basis of a multiscan spectrum. *f*) Hepatic *Hamp* levels were determined by quantitative PCR (n = 4 mice, respectively). *g*) Western blot analysis of whole liver lysates with specific antibodies to FPN and the control GAPDH (n = 3 mice, respectively). Densitometric analysis of FPN relative to GAPDH protein and one of 3 representative western blot experiments are shown. Statistical analysis was performed with IBM SPSS statistics 22. Data were compared with independent Student’s *t-*test. Values are expressed as the mean ± SEM, and statistically significant differences are indicated. *P< .05; **P< .01; ***P< .001; ns, not significant

To gain a better understanding of the signaling mechanism by which SpvB regulates hepcidin expression, we examined both the BMP/SMAD and JAK/STAT3 pathways in the liver of WT-infected and *ΔspvB*-infected mice at 3 days post-infection. The protein level of SMAD1/5/9 phosphorylation was increased in *S. typhimurium*-infected mice compared with the control group, while there were no apparent differences in pSMAD1/5/9 expression between mice infected with the WT and *ΔspvB* strains. However, mice infected with the WT strain showed a significantly higher level of STAT3 phosphorylation as compared to mice infected with the *ΔspvB* strain (Figure 3a, b). To further examine the observation that SpvB might interfere with systemic iron metabolism via facilitating STAT3 activation, *S. typhimurium*-infected mice were intraperitoneally injected with the STAT3 inhibitor Stattic. Our data showed that inhibiting STAT3 with Stattic eliminated the significant difference in the hepatic bacterial load between mice infected with the WT and *ΔspvB* strains (Figure 3c and Supplemental Fig. S1*A*). Importantly, it was found that the significant difference in serum iron concentration and hepatic iron content associated with the *Salmonella* effector SpvB were abrogated after Stattic treatment (Figure 3d, e). In line with these observations, the WT-infected mice showed a similar expression level of both hepcidin and FPN as compared with the *ΔspvB-*infected mice following Stattic treatment (Figure 3f, g). These data indicated that SpvB induced hepatic hepcidin expression through a STAT3-dependent manner, resulting in alternation of host iron metabolism and severe *Salmonella* pathogenesis.

### *SpvB causes an increase in hepatic inflammation following* S. typhimurium *infection*

**Figure 4.**
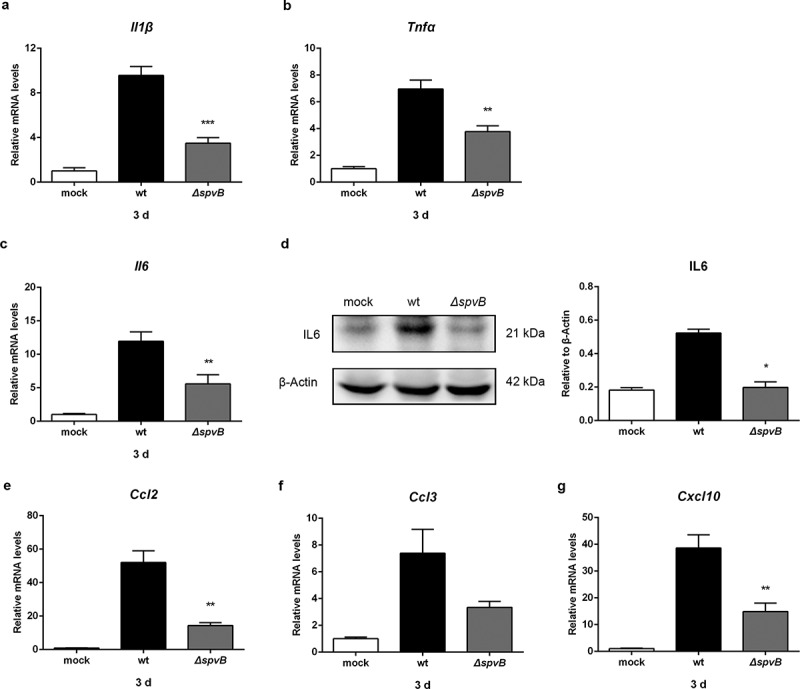
**SpvB promotes hepatic proinflammatory cytokine and chemokine expression in *S. typhimurium* infection**. Streptomycin-pretreated mice were orally infected with 1 × 10^7^ CFUs of either the WT or the *ΔspvB* mutant *S. typhimurium* strain and analyzed at 3 days post-infection. *a-c*) Hepatic *Il1β* (*a), Tnfα* (*b*) and *Il6* (*c*) levels were determined by quantitative PCR (n = 4 mice, respectively). *d*) Western blot analysis of whole liver lysates with specific antibodies to IL6 and the control β-Actin (n = 3 mice, respectively). Densitometric analysis of IL6 relative to β-Actin protein and one of 3 representative western blot experiments are shown. *e-g*) Hepatic *Ccl2* (*e), Ccl3* (*f*) and *Cxcl10* (*g*) levels were determined by quantitative PCR (n = 4 mice, respectively). Statistical analysis was performed with IBM SPSS statistics 22. Data were compared with independent Student’s *t-*test. Values are expressed as the mean ± SEM, and statistically significant differences are indicated. *P< .05; **P< .01; ***P< .001

Since the efficacy of antimicrobial immune responses is intimately linked to iron metabolism, we subsequently examined the influence of SpvB on the levels of cytokines that are known to be important for host resistance to intracellular pathogens. The mRNA levels of interleukin 1β (IL1β) and tumor necrosis factor alpha (TNFα) in the liver of WT-infected mice were significantly higher than those in the liver of *ΔspvB*-infected mice at 3 days post-infection (Figure 4a, b). Importantly, interleukin 6 (IL6), the primary inducer of STAT3-mediated hepcidin synthesis^23,24^ was significantly increased in the liver of WT-infected mice as compared to *ΔspvB*-infected mice at 3 days post-infection (Figure 4c, d). These findings further confirmed our observation that SpvB regulates STAT3 activation.

**Figure 5.**
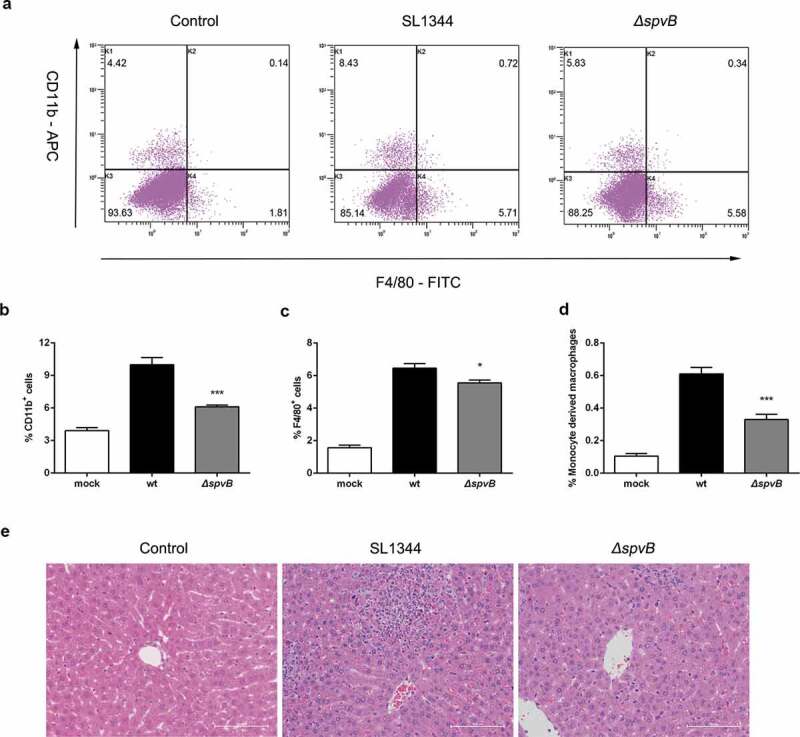
**SpvB increases inflammatory cell infiltration following *S. typhimurium* infection**. Streptomycin-pretreated mice were orally infected with 1 × 10^7^ CFUs of either the WT or the *ΔspvB* mutant *S. typhimurium* strain and analyzed at 3 days post-infection. *a*) Flow cytometric dot plots of hepatic non-parenchymal cells (n = 5 mice, respectively). *b-d*) Percentage of liver-infiltrated cell populations in *S. typhimurium-*infected mice. *e*) Histopathological analysis of the liver. Original magnification, ×20; scale bars: 100 μm. One of 3 representative histology experiments is shown. Statistical analysis was performed with IBM SPSS statistics 22. Data were compared with independent Student’s *t-*test. Values are expressed as the mean ± SEM, and statistically significant differences are indicated. *P< .05; ***P< .001

We further presumed that the *Salmonella* effector SpvB may be associated with inflammatory cells infiltration. At 3 day post-infection, higher mRNA levels of C-C motif chemokine ligand 2 (CCL2), C-C motif chemokine ligand 3 (CCL3) and C-X-C motif chemokine ligand 10 (CXCL10) were found in the liver of mice infected with the WT strain as compared to mice infected with the *ΔspvB* strain (Figure 4e–g). As a complementary approach, non-parenchymal cells isolated from the livers of WT-infected mice and *ΔspvB*-infected mice were analyzed by flow cytometry to confirm the observed effects of SpvB on inflammatory cell recruitment (Figure 5a). At 3 days post-infection, mice infected with the WT strain displayed a higher number of hepatic non-parenchymal cells expressing the pan-myeloid marker CD11b than mice infected with the *ΔspvB* strain (Figure 5b). Similar to this observation, more F4/80-positive non-parenchymal cells were found in the liver of WT-infected mice as compared with *ΔspvB*-infected mice (Figure 5c). Liver-associated F4/80^+^CD11b^+^ non-parenchymal cells are inflammatory monocyte-derived macrophages. Our data showed that mice infected with the WT strain contained a significantly higher number of hepatic F4/80^+^CD11b^+^ cells than mice infected with the *ΔspvB* strain at 3 days post-infection (Figure 5d). Additionally, the histopathological appearance of the liver showed severe inflammatory responses, such as more inflammatory cells around the hepatocytes, in mice infected with the WT strain as compared to mice infected with the *ΔspvB* strain (Figure 5e). These data suggested that SpvB contributes to an increased inflammatory response, a potential cause of iron metabolic disorder, in the liver during *S. typhimurium* infection.

### SpvB-mediated iron metabolic disorder is ameliorated by the TREM-1 inhibitor LP17

**Figure 6.**
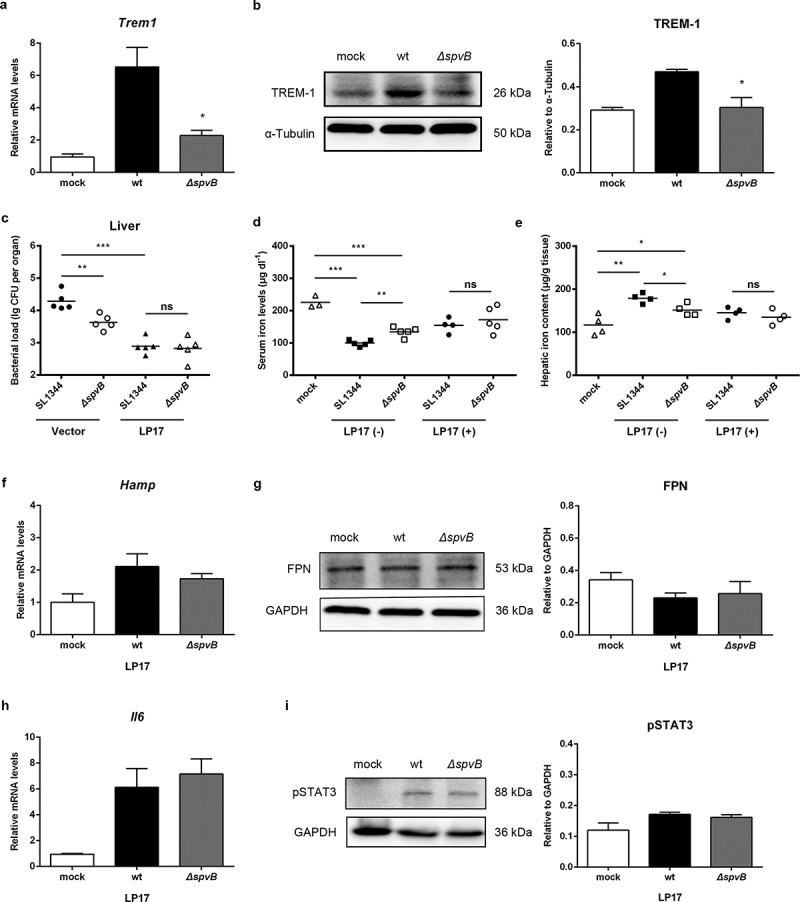
**SpvB-mediated iron metabolic disorder is ameliorated by the TREM-1 inhibitor LP17**. *a, b*) Streptomycin-pretreated mice were orally infected with 1 × 10^7^ CFUs of either the WT or the *ΔspvB* mutant *S. typhimurium* strain and analyzed at 3 days post-infection. *a*) Hepatic *Trem1* levels were determined by quantitative PCR (n = 4 mice, respectively). *b*) Western blot analysis of whole liver lysates with specific antibodies to TREM-1 and the control α-Tubulin (n = 3 mice, respectively). Densitometric analysis of TREM-1 relative to α-Tubulin protein and one of 3 representative western blot experiments are shown. *c-i*) *S. typhimurium*-infected mice were administered i.p with either LP17 or the same volume of vector and analyzed at 3 days post-infection. *c*) Hepatic bacterial load was determined by plating. *d*) Serum iron levels were measured with a colorimetric assay. *e*) Hepatic iron content was determined on the basis of a multiscan spectrum. *f*) Hepatic *Hamp* levels were determined by quantitative PCR (n = 4 mice, respectively). *g*) Western blot analysis of whole liver lysates with specific antibodies to FPN and the control GAPDH (n = 3 mice, respectively). Densitometric analysis of FPN relative to GAPDH protein and one of 3 representative western blot experiments are shown. *h*) Hepatic *Il6* levels were determined by quantitative PCR (n = 4 mice, respectively). *i*) Western blot analysis of whole liver lysates with specific antibodies to pSTAT3 and the control GAPDH (n = 3 mice, respectively). Densitometric analysis of pSTAT3 relative to GAPDH protein and one of 3 representative western blot experiments are shown. Statistical analysis was performed with IBM SPSS statistics 22. Data were compared with independent Student’s *t-*test. Values are expressed as the mean ± SEM, and statistically significant differences are indicated. *P< .05; **P< .01; ***P< .001; ns, not significant

It has been demonstrated that triggering receptor expressed on myeloid cells 1 (TREM-1), an amplifier of inflammation that has limited expression in healthy livers, but upregulated expression during bacterial infections and liver injury, plays a fundamental role in promoting proinflammatory cytokine and chemokine secretion, as well as inflammatory cell infiltration.^20^ Thus, we presumed that TREM-1 may be involved in SpvB-mediated increased hepatic inflammation and *Salmonella* virulence. In line with our hypothesis, TREM-1 expression at both the mRNA and protein levels were significantly higher in the livers of WT-infected mice than *ΔspvB*-infected mice at 3 days post-infection (Figure 6a, b). To investigate this possibility further, WT-infected mice and *ΔspvB*-infected mice were intraperitoneally injected with the TREM-1 inhibitor LP17. At 3 days post-infection, LP17 treatment reversed the significant difference in the hepatic bacterial burden in mice infected with the WT strain as compared to mice infected with the *ΔspvB* strain (Figure 6c and Supplemental Fig. S1*B*).

We subsequently investigated the potential role of TREM-1 in SpvB perturbing host iron homeostasis. At 3 days post-infection, *S. typhimurium*-infected mice receiving LP17 presented a less severe dysregulation of iron metabolism as evidenced by an increased serum iron level and reduction in hepatic iron content. LP17 administration also abrogated the effects of SpvB on host iron homeostasis (Figure 6d, e). In agreement, mice intraperitoneally injected with LP17 reversed the significant difference in hepatic hepcidin expression and FPN protein level between these two groups of *S. typhimurium*-infected mice (figure 6f, g). Moreover, data showed that LP17 not only abrogated the significant difference in IL1β and TNFα expression, but also eliminated the apparent difference in CCL2 and CXCL10 expression between both groups of *S. typhimurium*-infected mice at 3 days post-infection (Supplemental Fig. S1*C-G*). These results indicated that TREM-1 contributes to SpvB-mediated inflammatory response. We further investigated whether LP17 influences the role of SpvB in activating IL6/JAK/STAT3 signaling. Indeed, both IL6 expression and STAT3 phosphorylation were similar in the livers of WT-infected mice as compared to *ΔspvB*-infected mice following LP17 treatment (Figure 6h, Figure 6i and Supplemental Fig. S1*H*). Taken together, these data demonstrated that inhibiting TREM-1 expression was beneficial to alleviating *Salmonella* pathogenesis and dysregulation of host iron homeostasis caused by the *Salmonella* effector SpvB.

### SpvB interferes iron metabolism when macrophages co-cultured with hepatocytes

**Figure 7.**
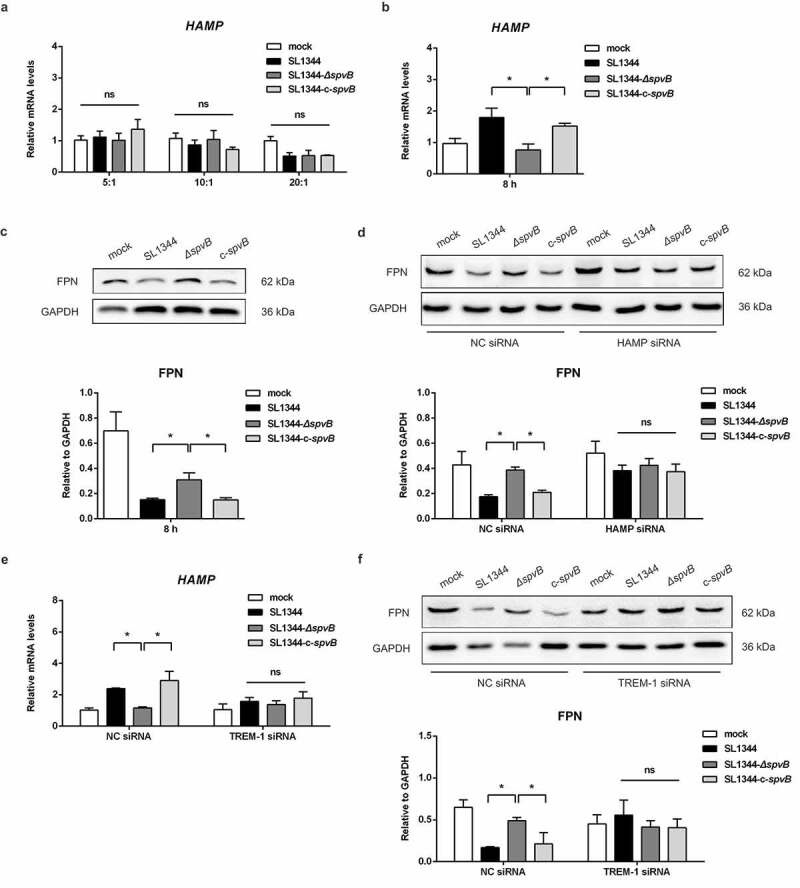
**SpvB interferes macrophage iron metabolism when interacted with hepatocytes**. *a*) HepG2 cells were infected with the WT, *ΔspvB* or c-*spvB S. typhimurium* strain at an MOI of 5, 10 or 20 for 8 h. *HAMP* levels were determined by quantitative PCR. *b, c*) Co-culture cells were infected with the WT, *ΔspvB* or c-*spvB S. typhimurium* strain at an MOI of 10 for 8 h. *b*) *HAMP* levels in co-cultured HepG2 cells were determined by quantitative PCR. *c*) Western blot analysis of co-cultured THP-1 macrophages lysates with specific antibodies to FPN and the control GAPDH. Densitometric analysis of FPN relative to GAPDH protein and one of 3 representative western blot experiments are shown. *d*) HepG2 cells with or without silencing of *HAMP* were co-cultured with THP-1 macrophages and then infected with the WT, *ΔspvB* or c-*spvB S. typhimurium* strain at an MOI of 10 for 8 h. Western blot analysis of co-cultured THP-1 macrophages lysates with specific antibodies to FPN and the control GAPDH. Densitometric analysis of FPN relative to GAPDH protein and one of 3 representative western blot experiments are shown. *e, f*) THP-1 cells with or without silencing of *TREM-1* were co-cultured with HepG2 cells and then infected with the WT, *ΔspvB* or c-*spvB S. typhimurium* strain at an MOI of 10 for 8 h. *e*) *HAMP* levels in co-cultured HepG2 cells were determined by quantitative PCR. *f*) Western blot analysis of co-cultured THP-1 macrophages lysates with specific antibodies to FPN and the control GAPDH. Densitometric analysis of FPN relative to GAPDH protein and one of 3 representative western blot experiments are shown. Statistical analysis was performed with IBM SPSS statistics 22. The data were compared by ANOVA with Student-Newman-Keuls (S-N-K) correction. Values are expressed as the mean ± SEM of three independent experiments, and statistically significant differences are indicated. *P< .05; ns not significant

To further explore the role of SpvB in regulating hepcidin-FPN axis, we investigated whether SpvB could directly influence the transcription of hepcidin. As shown in Figure 7a, there were no apparent differences in *HAMP* expression among HepG2 cells infected with the WT, *ΔspvB*, or c-*spvB* strain. Subsequently, a HepG2 cells/THP-1 macrophages co-culture model was established, and we found that cells infected with the WT or the c-*spvB* strain displayed a higher mRNA transcript level of hepcidin as compared to those infected with the *ΔspvB* strain (Figure 7b). Meanwhile, FPN protein level was decreased more significantly in the WT or the c-*spvB*-infected cells than in the *ΔspvB*-infected cells (Figure 7c). Importantly, siRNA-mediated downregulation of *HAMP* in co-cultured HepG2 cells abrogated the significant difference in FPN expression among these *Salmonella*-infected THP-1 macrophages (Figure 7d and Supplemental Fig. S1*I*). These observations indicated that cytokines such as IL6 produced by THP-1 cells were required for up-regulation of hepcidin transcription in HepG2 cells. To further investigate whether the dysregulation of systemic iron metabolism is directly caused by the effect of SpvB, THP-1 macrophages transfected with pEGFP-*spvB* or the control vector pEGFP-N1 were co-cultured with HepG2 cells with or without silencing of *HAMP*. As shown in Supplemental Fig. S1*J*, THP-1 macrophages transfected with pEGFP-*spvB* contained a significantly higher cellular iron concentration than those transfected with the control pEGFP-N1 vector. Importantly, siRNA-mediated downregulation of *HAMP* in co-cultured HepG2 cells resulted in a reduction of cellular iron content in THP-1 macrophages transfected with pEGFP-*spvB*, indicating that SpvB is *per se* to modulate hepcidin–FPN axis and regulate host systemic iron metabolism. In addition, when co-cultured with HepG2 cells with silencing of *HAMP*, we also found that iron load of THP-1 macrophages transfected with pEGFP-*spvB* was still higher than those transfected with the control pEGFP-N1 vector. This observation was in consistent with our previous observation that SpvB is *per se* to modulate cellular iron metabolism.^15^

TREM-1 has been characterized as a receptor expressed on macrophages rather than hepatocytes.^20^ *TREM-1* knockdown in co-cultured THP-1 macrophages could reverse the effect of SpvB on hepcidin and FPN (Figure 7e,f and Supplemental Fig. S1*K*). These data further verified our results obtained *in vivo* that SpvB regulates the hepcidin–FPN axis in a TREM-1-dependent manner, and targeting TREM-1 may provide potential therapeutics for treating salmonellosis.

## Discussion

**Figure 8.**
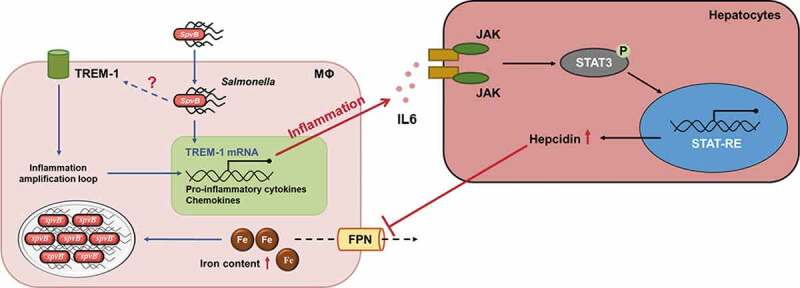
**Model of SpvB in interfering with systemic iron metabolism**. SpvB, an important pSLT-encoded cytotoxic effector, contributes to extensive and severe inflammation during *S. typhimurium* infection through activation of TREM-1 in macrophages. This subsequently induces hepatocyte hepcidin transcription via a IL6/JAK/STAT3-dependent manner. The induction of hepcidin expression in turn inhibits the sole iron exporter FPN, then results in an increased intramacrophage iron concentration and further facilitates hypoferremia and hepatic iron accumulation, which ultimately benefits for *Salmonella* survival and replication. MΦ, macrophage

Host iron homeostasis is tightly controlled by cellular- and systemic-dependent regulatory systems.^10^ Our previous study showed that SpvB plays an important role in promoting *Salmonella* virulence by facilitating *Salmonella* survival and proliferation within macrophages via interfering with cellular iron metabolism. However, the mechanisms of SpvB underlying *Salmonella*-relevant dysregulation of systemic iron metabolism have not been thoroughly studied. Here, we provided the first evidence, to our knowledge, that SpvB is one of the major participants of *Salmonella*-induced hepcidin expression leading to disorders of host iron homeostasis. We showed that the IL6/JAK/STAT3 pathway, but not the BMP/SMAD-dependent pathway, was involved in the SpvB-mediated upregulation of hepatic hepcidin and downregulation of FPN, which resulted in severe hypoferremia and strong iron accumulation in the liver. Moreover, SpvB promoted hepatic TREM-1 activation, thus facilitating secretion of proinflammatory cytokines and chemokines, as well as infiltration of inflammatory cells. Treatment of *S. typhimurium-*infected mice with the TREM-1 inhibitor LP17 was advantageous for ameliorating SpvB-mediated hepatic inflammation and, therefore, iron metabolic disorder (Figure 8).

Iron is an essential molecule in many biological processes, such as the host antimicrobial immune response.^8^ During *Salmonella* infection, iron limitation is considered to be a central component of nutritional immunity.^25^ Since the intracellular growth and replication of *Salmonella* is closely related to a sufficient iron supply, restriction in the availability of this essential nutrient metal can protect the host from severe symptoms. In response to changes in iron concentration within the microenvironment, we hypothesized that *Salmonella* may also employ strategies, especially the virulence factors located on the pSLT virulence plasmid, to circumvent the host iron limitation *in vivo*. To address this, we investigated the alternation of the host iron metabolism after oral infection of mice with the WT or *ΔspvB S. typhimurium* strain. Our results in this work, which demonstrated an increased hepatic bacterial burden associated with decreased serum iron content, were in line with our previous observation, which reported enhanced *S. typhimurium* pathogenesis and severe hypoferremia caused by SpvB. To our surprise, SpvB contributed to a significantly higher hepatic iron concentration and splenic iron overload in mice orally infected with *S. typhimurium* (data not shown), which was inconsistent with our previous research that demonstrated a lower splenic iron content attributed to SpvB in an intraperitoneal infection mouse model. It has been reported that mice infected with *Salmonella* through oral or intraperitoneal administration might cause divergent outcomes of host iron metabolism. For example, intraperitoneal infection of mice with *S. typhimurium* led to an increase in FPN expression, however, oral-infected mice showed a decrease in FPN level^9,17,26^ We speculated bacterial burden and their utilization of iron might be involved in differences of tissue iron concentration between oral and intraperitoneal administration.

Given that the hepcidin-FPN axis is believed to be a key regulator in the systemic regulation of iron metabolism, we presumed this essential iron regulatory axis was involved in the SpvB-mediated iron metabolic disorder within the liver. In support of this hypothesis, SpvB resulted in an increased expression of hepcidin, which was associated with a reduction in FPN protein levels. A previous study showed that hepcidin-deficient mice were more susceptible to *S. typhimurium* infection following intravenous administration.^27^ Recently, a study from another laboratory reported that hepcidin deficiency had minimal effects on the growth of *S. typhimurium* in an intravenous infection mouse model.^28^ However, our current data showed that there was a significant decrease of hepatic bacterial loads in *Hamp^−/−^* mice when compared to WT mice following oral infection, suggesting that hepcidin is one of the harmful factors involved in host resistance to *S. typhimurium*. In addition to this observation, we found that oral infection of *Hamp^−/−^* mice with the WT strain did not elicit apparent differences in the hepatic bacterial burden as compared to mice infected with the *ΔspvB* strain. These data suggested a role of SpvB in perturbing systemic iron metabolism by modulating the hepcidin–FPN axis. Taken together with our previous findings on SpvB interfering with cellular iron metabolism, we revealed that SpvB is an effective strategy utilized by *Salmonella* to scavenge iron from the host in order to achieve their own nutrient requirements.

To identify SpvB might act directly on hepatocytes to induce hepcidin transcription, either HepG2 cells only or HepG2 cells/THP-1 macrophages co-cultured model was exposed to the WT, *ΔspvB* or c-*spvB* strain. Interestingly, the hepcidin up-regulating activity caused by SpvB occurred in the presence of macrophages. During bacterial infections and inflammatory conditions, hepcidin transcription responds to a variety of inflammatory signals and mediators^23,29^ Our *in vitro* results supported these previous observations showing that cytokines secreted by macrophage play an integral role in altering hepcidin expression. Using ectopic expressed SpvB in co-cultured THP-1 macrophages, we confirmed that SpvB is *per se* to regulate systemic iron metabolism via modulating the hepcidin–FPN axis. Taken together, our previous publication showed that SpvB-mediated dysregulation of macrophage iron metabolism was present in early infection stage.^15^ In current study, we further observed that SpvB could perturb macrophage iron homeostasis in late infection stage via regulating the hepcidin–FPN axis.

There are two major signaling pathways that are known to regulate hepcidin transcription in the liver and hence systemic iron metabolism: one is the BMP/SMAD pathway and the other is the JAK/STAT3 pathway.^30^ In this study, our data showed that SpvB caused a significantly higher level of STAT3 phosphorylation, while no apparent difference was found in SMAD1/5/9 phosphorylation. Inhibition of STAT3 not only ameliorated symptoms in disorders of iron homeostasis but also reduced the hepatic bacterial burden that was mediated by SpvB. These observations demonstrated that the STAT3-dependent pathway, but not the BMP/SMAD pathway, was involved in the process of SpvB-mediated hepcidin synthesis. It has been suggested that the stimulus for the JAK/STAT3 pathway is primarily inflammatory cytokines. Among them, IL6, rather than cytokines associated with the type I response, such as TNFα, induces a signaling cascade that leads to the phosphorylation of STAT3.^31^ Phosphorylated STAT3 subsequently translocates to the nucleus and promotes the transcription of hepcidin^32,33^ In line with our results regarding SpvB-induced STAT3 phosphorylation, a significant reduction in the expression of IL6 was found in mice infected with the *ΔspvB* strain as compared to those infected with the WT strain.

TREM-1, a cell-surface-activating receptor expressed on macrophages and monocytes in the liver, is elevated during bacterial challenge.^34^ TREM-1 is considered to be an important therapeutic target in several inflammatory diseases, such as severe sepsis and pneumonia^35,36^ A recent publication reported a new function of TREM-1 in intensifying hepatic inflammation and fibrogenesis.^20^ However, to the best of our knowledge, it is not completely understood if TREM-1 signaling participates in *Salmonella*-relevant disorders of systemic iron metabolism. In this study, we showed that SpvB resulted in a significantly higher expression of TREM-1 and increased proinflammatory cytokine production such as CCL2, which was paralleled by an enhanced infiltration of inflammatory cells and hepatocellular damage in the liver. Our inhibitor assays also indicated that hepatic iron dysregulation was downstream of TREM-1 activation. When TREM-1 expression was inhibited by LP17, hypoferremia and hepatic iron accumulation caused by SpvB were abrogated. Importantly, similar *S. typhimurium* loads were observed in the liver of WT- and *ΔspvB*-infected mice after LP17 treatment, which suggested that targeting TREM-1 might provide potential therapeutic strategies to prevent or alleviate *Salmonella* pathogenesis. Furthermore, consistent with our results obtained *in vivo, TREM-1* knockdown in macrophages could abrogate the effects of SpvB on hepcidin-FPN axis in hepatocytes/macrophages co-cultured system. However, even though the relationship between SpvB and TREM-1 has been demonstrated in this work, further investigation is required to identify the underlying mechanism. It has been reported that inhibition of the proteasome suppresses TREM-1 expression^37,38^ Interestingly, our previous study demonstrated that proteasome pathways were involved in SpvB-relevant salmonellosis. However, whether SpvB activated TREM-1 expression via the proteasome pathway remains to be further discussed.

In summary, our findings further extended the knowledge regarding the SpvB-associated pathogenesis of *Salmonella*. As an extension of our previous study where we showed that SpvB disturbed cellular iron metabolism, in this study we revealed a novel contribution of SpvB to *Salmonella*-induced disorders of systemic iron metabolism by controlling the hepcidin-FPN axis. By activating TREM-1 signaling, SpvB contributes to extensive and severe hepatic inflammation. This subsequently induces hepcidin expression in an IL6/JAK/STAT3-dependent manner, and eventually interfering with the host nutritional immune strategy of iron withhold.

## Supplementary Material

Supplemental Material

## Figures and Tables

**Table:** 

*Salmonella enterica* serovar Typhimurium	*S. typhimurium*
*Salmonella* plasmid virulence	*spv*
*Salmonella*-containing vacuole	SCV
*Salmonella* pathogenicity island-2	SPI-2
type III secretion system	T3SS
hepcidin	HAMP
ferroportin	FPN
wild-type	WT
deferasirox	DFX
janus kinase	JAK
signal transducer and activator of transcription	STAT
bone morphogenic protein	BMP
small mothers against decapentaplegic	SMAD
interleukin 1β	IL1β
tumor necrosis factor alpha	TNFα
interleukin 6	IL6
C-C motif chemokine ligand 2	CCL2
C-C motif chemokine ligand 3	CCL3
C-X-C motif chemokine ligand 10	CXCL10
triggering receptor expressed on myeloid cells 1	TREM-1
Luria-Bertani	LB
colony-forming units	CFUs
hematoxylin-eosin	H&E
Hank’s Balanced Salt Solution	HBSS
*spvB* deletion mutant strain	*ΔspvB*
multiplicity of infection	MOI
