# Supplementary material for: Salmonella effector SpvB aggravates dysregulation of systemic iron metabolism via modulating the hepcidin−ferroportin axis
Source: Gut Microbes. 2021 Jan 21;13(1):1849996. doi: 10.1080/19490976.2020.1849996 (PMC7833757; doi:10.1080/19490976.2020.1849996)
Supplement: Supplemental Material [file KGMI_A_1849996_SM5647.zip › Supplementary information/Supplementary Material - For review.docx]

**Supplemental Figure 1.**

*A-H*) Streptomycin-pretreated mice were orally infected with 1*10^7^ CFUs of either the WT or the *ΔspvB* mutant *S. typhimurium* strain. *A*) *S. typhimurium*-infected mice were administered i.p with Stattic and analyzed at 3 days post-infection. Western blot analysis of whole liver lysates, with specific antibodies to pSTAT3 and the control GAPDH (n = 5 mice, respectively). *B*-*H*) *S. typhimurium*-infected mice were administered i.p with LP17 and analyzed at 3 days post-infection. *B*) Western blot analysis of whole liver lysates, with specific antibodies to TREM-1 and the control α-Tubulin (n = 5 mice, respectively). Hepatic *Il1β* (*C*), *Tnfα* (*D*), *Ccl2* (*E*), *Ccl3* (*F*) and *Cxcl10* (*G*) levels were determined by quantitative PCR (n = 4 mice, respectively). *H*) Western blot analysis of whole liver lysates, with specific antibodies to IL6 and the control β-Actin (n = 5 mice, respectively). *I*) *HAMP* gene expression on HepG2 cells with or without silencing of *HAMP* was determined by quantitative PCR. *J*) THP-1 macrophages transfected with pEGFP-*spvB* or the control pEGFP-N1 were co-cultured with HepG2 cells with or without silencing of *HAMP* for 12 h. The relative iron concentration in co-cultured macrophages was determined on the basis of a multiscan spectrum (n = 7, respectively). *K*) TREM-1 protein level on THP-1 cells with or without silencing of *TREM1* was determined by Western blot analysis. Statistical analysis was performed with IBM SPSS statistics 22. Data were compared with independent Student’s *t-*test. Values are expressed as the mean ± SEM, and statistically significant differences are indicated. ^**^*P*<0.01.
