# Supplementary figures and images for: Salmonella effector SpvB aggravates dysregulation of systemic iron metabolism via modulating the hepcidin−ferroportin axis
Source: Gut Microbes. 2021 Jan 21;13(1):1849996. doi: 10.1080/19490976.2020.1849996 (PMC7833757; doi:10.1080/19490976.2020.1849996)

# Supplement

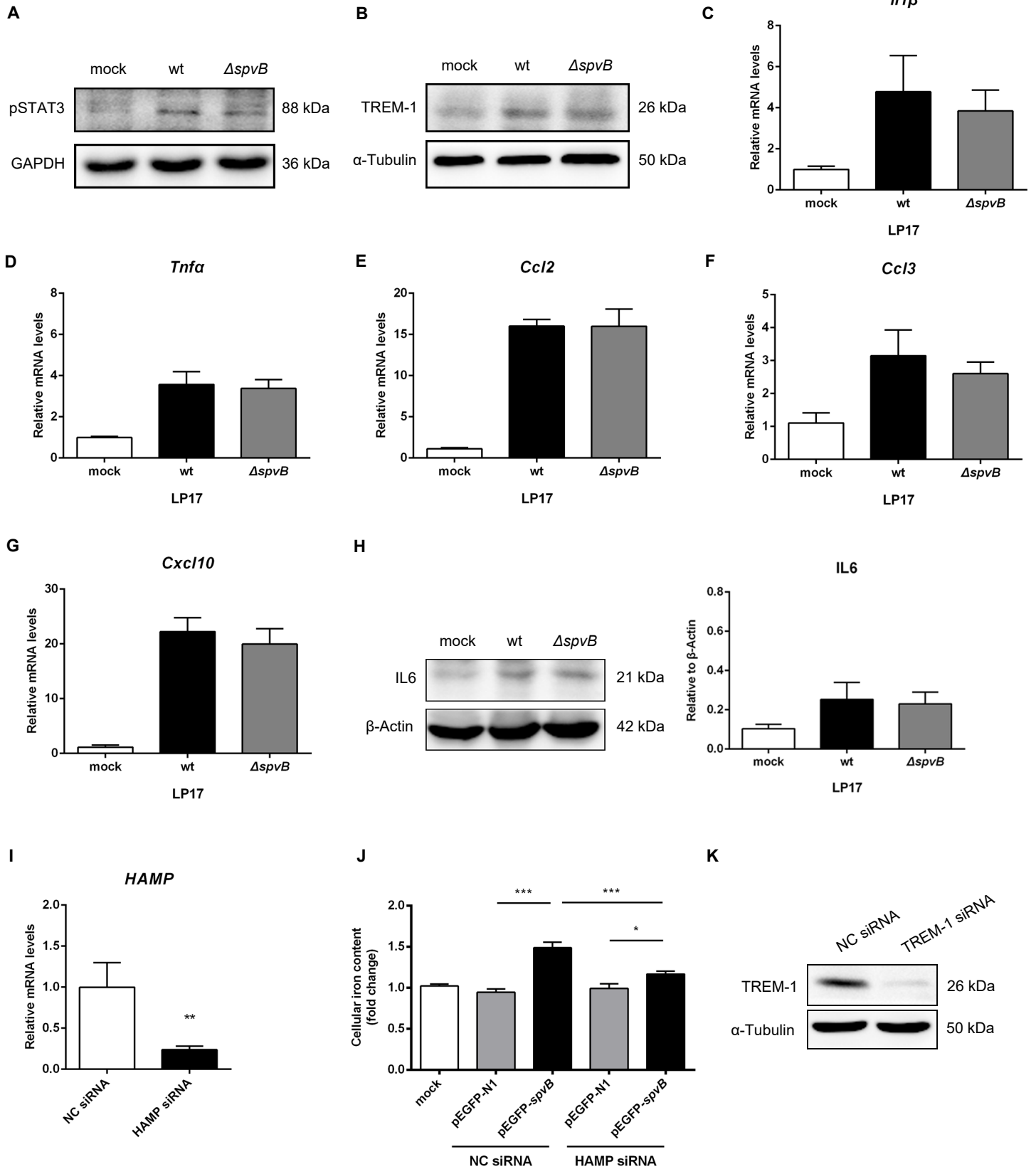

Supplement: Supplemental Material [file KGMI_A_1849996_SM5647.zip › Supplementary information/supplement.pdf]
